# *Rhinacanthin C* Ameliorates Insulin Resistance and Lipid Accumulation in NAFLD Mice via the AMPK/SIRT1 and SREBP-1c/FAS/ACC Signaling Pathways

**DOI:** 10.1155/2023/6603522

**Published:** 2023-01-10

**Authors:** Zhiqiang Gong, Sha Han, Chenlin Li, Tianxiu Meng, Yu Huo, Xianfu Liu, Yanhong Huang, Lifang Yang

**Affiliations:** ^1^School of Chemistry and Chemical Engineering, Guangxi Minzu University, Guangxi Key Laboratory for Polysaccharide Materials and Modifications, Guangxi Key Laboratory of Chemistry and Engineering of Forest Products, Nanning, Guangxi, China; ^2^Faculty of Chinese Medicine Science, Guangxi University of Chinese Medicine, Guangxi Key Laboratory of Zhuang and Yao Ethnic Medicine Nanning, Nanning, Guangxi, China

## Abstract

*Rhinacanthin C* (RC) is a naphthoquinone ester with an anti-inflammatory activity extracted from *Rhinacanthus nasutus* (L.) Kurz (Rn). It has been proven to improve hyperglycemia and hyperlipidemia, but the prevention and mechanism of RC in nonalcoholic fatty liver disease (NAFLD) are not clear. In the current study, we first extracted RC from Rn using ethyl acetate and identified it by HPLC, MS, and NMR. At the same time, molecular docking analysis of RC with AMPK and SREBP-1c was performed using AutoDock software. In addition, the mouse model of NAFLD was induced by a high-fat diet in vivo, and low, medium, and high concentrations of RC were used for intervention. The results showed that RC significantly reduced the body mass and liver body coefficient of NAFLD mice, inhibited liver inflammation and fat accumulation, and improved insulin resistance. Further studies showed that RC significantly reduced the levels of serum leptin and resistin, upregulated the expression levels of adiponectin and adiponectin receptor in the liver, and inhibited the expression levels of MCP-1, TNF-*α,* and IL-6. In terms of mechanism, RC upregulates the expression of p-AMPK and SIRT1 and downregulates the expression of p-p65, SREBP-1c, Fas, Acc-*α*, PPAR-*γ*, and SCD1. These studies suggest that RC improves insulin resistance and lipid accumulation in NAFLD by activating the AMPK/SIRT1 and SREBP-1c/Fas/ACC pathways, respectively.

## 1. Introduction

Nonalcoholic fatty liver disease (NAFLD) is characterized by fat accumulation in hepatocytes. It ranges from simple steatosis (nonalcoholic fatty liver or NAFL) to nonalcoholic steatohepatitis (NASH) with inflammation, hepatocyte ballooning, and various levels of fibrosis and is associated with a significant risk of progression to liver cirrhosis and hepatocellular carcinoma [1]. The diagnosis of NAFLD must exclude hepatic viral infections, excessive alcohol intake, and the use of potentially hepatotoxic drugs [2]. As the leading chronic liver disease, NAFLD affects approximately 1.7 billion individuals worldwide, and its prevalence is on the rise due to the increase in overweight and obesity. In the United States, the number of NAFLD cases is expected to increase from 83.1 million in 2015 to 100.9 million in 2030 [3], which will continuously increase health and economic burdens. In addition, a recent meta-analysis found that the prevalence of NAFLD in Asian countries is similar to that in Western countries, with the highest incidence rate and incidence and NAFLD-related mortality rate in China [4]. Currently, no pharmacologic therapy is approved for the treatment of NAFLD, except for lifestyle changes based on weight and exercise.

The mechanisms leading to NAFLD are unclear to date. The two-hit hypothesis has been proposed to explain the pathogenesis of NAFLD. The first hit is insulin resistance and excessive fatty acids in the circulation, which leads to simple hepatic steatosis. Insulin resistance is pivotal for the progression of NAFLD, as 70%–80% of obese and diabetic patients have NAFLD [5]. Studies have shown that hyperinsulinemia and dyslipidemia are more severe in obese patients with NAFLD than in those without NAFLD [6]. The liver, which is responsible for the synthesis, export, and subsequent redistribution of new fatty acids, is also an essential organ for lipid metabolism [7]. These processes are closely regulated by complex interactions between nuclear receptors, transcription factors, and hormones so that the liver lipid homeostasis is strictly controlled [8]. The disruption of one or more of these processes may precipitate the retention of fat within the liver and the subsequent development of NAFLD. In addition, excess fatty acids circulate and accumulate in peripheral tissues, including the liver and adipose tissue, further leading to insulin resistance [9].

*Rhinacanthus nasutus* (L.) Kurz (Rn), generally referred to as “Bai-He-Ling-Zhi” in China, is native to Southeast Asian countries and belongs to the family Acanthaceae. Its pharmaceutical preparations are taken orally in the form of a decoction or herbal tea to treat hepatitis, diabetes, and hypertension [10]. The constituents of therapeutic value found in Rn are naphthoquinones, which have been reported to display inhibitory acetylcholinesterase, antidiabetic, and anti-inflammatory properties [11]. *Rhinacanthin C* (RC) is the main naphthoquinone component (approximately 62.2%) in standardized Rhinacanthin-rich extract and has been shown to have a protective effect on diabetic nephropathy, and its mechanism is achieved by alleviating oxidative stress and the inflammatory response in the kidney [12]. However, the protective effects of RC on hepatic insulin resistance and lipid accumulation have yet to be reported. Therefore, this study evaluated the ability of RC to inhibit the progression of NAFLD.

## 2. Materials and Methods

### 2.1. Materials

A normal diet was purchased from Jiangsu Synergetic Pharmaceutical Bioengineering Co., Ltd. (Nanjing, Jiangsu, China, XTADM001) and consisted of 4% fat, 73.1% carbohydrate, and 14.2% protein. A high-fat diet was purchased from Jiangsu Synergetic Pharmaceutical Bioengineering Co., Ltd. (Nanjing, Jiangsu, China, XTHF60) and consisted of 60% fat, 20% carbohydrate, and 20% protein. Metformin was purchased from Sigma (St. Louis, MO, USA, D150959-5G). Hematoxylin eosin (H&E) and oil red O (ORO) staining kits were purchased from Solarbio (Beijing, China, G1120 and G1261). The rabbit antimouse adiponectin (ADIPOQ), MCP-1, TNF-*α*, IL-6, and DAB horseradish peroxidase chromogenic kits were purchased from Beyotime (Shanghai, China). The rabbit antimouse adiponectin receptor II (ADIPOR2), phospho-AMPK*α*1, AMPK*α*1, SIRT1, phospho-NF-*κ*B p65, NF-*κ*B p65, SREBP1C, FAS, acetyl coenzyme A carboxylase (ACC), PPAR-*γ*, SCD1, *β*-actin, and goat antirabbit IgG antibodies were purchased from Abcam (Cambridge, UK).

### 2.2. Plant Material

The fresh grass of Rn was collected from the plant botanical garden of Guangxi University of Chinese Medicine (Guangxi Nanning, China) and was certified by *Prof.* Xie Lisa, the School of Pharmacy of Guangxi University of Chinese medicine, where herbarium specimens (Voucher No. 20210305001) are kept.

### 2.3. Isolation of *Rhinacanthin C*

The dried powder of Rn (5 kg) was refluxed by ethyl acetate (20 L × 3), and the pooled extracts of the same solvent were concentrated under reduced pressure to obtain 80.6 g of extract. Ion-exchange chromatography was used to separate and purify *Rhinacanthin C* [13]. The extract was dissolved in methanol, filtered, stirred with 2 kg of activated Amberlite TRA-67 (Aladdin, Shanghai, China), and allowed to stand for 1 h. The treated resin was poured into a glass column (10 × 80 cm) and eluted with methanol (3 BV) until the green pigments were washed out. Then, 10% acetic acid methanol solution (3 BV) was added, and the entire solution was allowed to pass through the column at a flow rate of 1.5 mL/min. The eluent was evaporated to dryness under reduced pressure. Shimadzu LC-16p High Performance Liquid Chromatography (Shimadzu Technology, Tokyo, Japan) was used to separate the 10% acetic acid methanol eluent, which was then passed through a C18 chromatographic column (Cosmosil ODS-C18-ms-ii, 10 × 250 mm) with the mobile phase MeOH/0.2% TFA/H2O (82 : 18, v/v) at a flow rate of 4 mL/min. The quantification wavelength was set at 250 nm.

### 2.4. Molecular Docking

The crystal structures of AMPK*α* (No. 2ya3) and SIRT1 (No. 5btr) were obtained from the Protein Data Bank (PDB, https://www.rcsb.org/pbd/home/home.do). The docking analysis between RC and the protein receptor was carried out using AutoDock (Version: 4.2.6, Scripps Research, La Jolla, CA, USA), and AutoDock Vina 1.5.6 software developed by Olson's research group in Scripps Research Institute was adopted to assess molecular docking [14] and GROMACS (Version: 2021.1, University of Virginia, Charlottesville, VA, USA) software. The Lamarckian genetic algorithm was used for the docking algorithm. The binding energy between the small molecule and protein receptor was calculated, and the dominant conformation of the small molecule and the binding mode of the protein target was also observed and analysed.

### 2.5. Animal Experiment

Sixty C57BL/6JNifdc male wild-type mice (18∼20 g, ∼6 weeks of age) were obtained from Vital River Laboratory Animal Technology Co., Ltd. (Beijing, China). All mice were maintained in ventilated cages under specific pathogen-free conditions with a 12 h light/dark cycle and free access to purified water and a standard diet at 26°C and a stable atmospheric pressure. After 1 week of diet adaptation, mice were divided randomly into the control group (normal diet), HFD group (high-fat diet), metformin group (HFD with metformin 250 mg/kg p.o.), low-dose RC group (HFD with RC 5 mg/kg p.o.), medium-dose RC group (HFD with RC 10 mg/kg p.o.), and high-dose RC group (HFD with RC 20 mg/kg p.o.). Mice received oral metformin or RC once a day for 12 weeks. During this period, the body weight and food intake of the mice were recorded every two weeks. After the intervention, an oral glucose tolerance test (OGTT) and an insulin tolerance test (ITT) were performed as previously described, followed by starvation for 12 h [15]. The mice were anaesthetized by intraperitoneal injection of 1% sodium pentobarbital (40 mg/kg), and the liver and blood were collected. Then, the mice were euthanized by intraperitoneal injection of 1% sodium pentobarbital (800 mg/kg). The liver samples were stored at −80°C for subsequent experiments. The serum was separated from the blood by centrifugation at 3000 rpm for 20 min and stored at 4°C.

### 2.6. Biochemical Analysis

Liver tissue samples were homogenized using a tissue homogenizer (Mini Beadbeater, BioSPec, Rockville, MD, USA). Homogenates were incubated at 4°C for 30 min and then centrifuged at 3500 rpm for 10 min. The levels of triglyceride (TG, A110-1-1), total cholesterol (TC, A111-1-1), high-density lipoprotein cholesterol (HDL-C, A112-1-1), low-density lipoprotein cholesterol (LDL-C, A113-1-1), alanine aminotransferase (ALT, C009-2-1), aspartate aminotransferase (AST, C010-2-1), insulin (H203-1-1), glucose (F006-1-1), nonesterified free fatty acids (NEFA, A042-2-1) in serum, and the supernatant of liver tissue samples were determined using respective kits (Nanjing Jiancheng Bioengineering Institute, Nanjing, Jiangsu, China) following the manufacturers' instructions. The liver index was calculated using the following formula: liver index (%) = liver weight (g)/body weight (g) × 100% [16].

### 2.7. Enzyme-Linked Immunosorbent Assay

Frozen liver homogenates were used for the estimation of liver levels of superoxide dismutase (SOD, A001-3-2), glutathione peroxidase (GSH-Px, A005-1-2), and malondialdehyde (MDA, A003-1-2) using respective kits (Nanjing Jiancheng Bioengineering Institute, Nanjing, Jiangsu, China) following the manufacturers' instructions. Meanwhile, the protein levels of leptin (MM-0622M2), resistin (MM-0552M2), MCP-1 (MM-0082M2), TNF-*α* (MM-0132M2), and IL-6 (MM-0163M2) in serum were also measured using the respective kits (MMBio, Yancheng, Jiangsu, China) following the manufacturers' instructions [17].

### 2.8. Histological Analysis

Liver tissue was fixed in 4% paraformaldehyde for 24 h. Then, the tissue was embedded in paraffin and sectioned to 5 *μ*m thickness for H&E staining and observation using an optical microscope (CX41, Olympus, Tokyo, Japan). For ORO staining, frozen liver samples at an optimal cutting temperature, −18°C (Sakura Finetek Co, Torrance, CA, USA), were cut to generate sections of 15 *μ*m thickness and fixed with 75% alcohol at room temperature for 15 minutes. Finally, the sections were stained with ORO for 1 h. Then, liver sections were counterstained with hematoxylin before microscopic observation [18].

### 2.9. Immunohistochemistry

Paraffin sections of liver tissues were deparaffinized and rehydrated, incubated with 3% hydrogen peroxide for 10 min to block endogenous peroxidase activity, and blocked with 10% goat serum for 30 min. Subsequently, the sections were incubated overnight at 4°C with ADIPOQ (1 : 50, AF6156), ADIPOR2 (1 : 250, ab231051), MCP-1 (1 : 100, AF7437), TNF-*α* (1 : 50, AF8208), and IL-6 (1 : 100, AF7236). The next day, the sections were incubated with a biotinylated secondary antibody (1 : 50, ab6721) at room temperature for 45 min. The signals were visualized by DAB staining, and the nuclei were counterstained with hematoxylin [19]. Positive staining presented a yellow colour under an optical microscope. The protein expression data were semiquantitatively analysed as integrated optical density (IOD) in the positive area of the microphotograph with the Image-Pro Plus software (Vision 6.0, Media Cybernetics, Rockville, MD, USA).

### 2.10. Western Blot Analysis

Protein was extracted from liver tissues. A BCA Protein Quantitative Kit (Wanleibio, Shenyang, Liaoning, China, WLA004a) was used to determine the protein concentration. The protein (100 *μ*g for each sample) was separated by 10% sodium dodecyl sulfate-polyacrylamide gel electrophoresis (SDS‒PAGE) and then transferred to a PVDF membrane (Millipore, Billerica, MA, USA). After blocking with 5% skim milk, the membrane was incubated with targeted primary antibodies against p-AMPK*α* (1 : 1000, ab92701), AMPK*α* (1 : 1000, ab32047), SIRT1 (1 : 1000, ab189494), p-p65 (1 : 1000, ab76302), p65 (1 : 1000, ab32536), SREBP-1C (1 : 500, ab28481), FAS (1 : 1000, ab133619), ACC (1 : 1000, ab109368), PPAR-*γ* (1 : 1000, ab178860), SCD1 (1 : 1000, ab236868), and *β*-actin (1 : 5000, ab6276) overnight at 4°C and then with secondary antibody (1 : 1000, ab6721) at room temperature for 1 hour. Finally, the signals were detected by adding enhanced chemiluminescence (ECL) substrate (Millipore, Billerica, MA, USA) and visualized by a Tanon 5200 Imaging Analysis System (Tanon, Shanghai, China). Relative protein levels were determined by densitometry analysis using the Image-Pro Plus software [20]. The relative protein expression was normalized to *β*-actin.

### 2.11. Quantitative Real-Time PCR Analysis

The total RNA was extracted from liver tissues using the TRIzol reagent (Thermo Fisher Scientific, Waltham, MA, USA) according to the manufacturer's instructions. The RNA samples were reverse transcribed into cDNA with the PrimeScript™ RT reagent kit (Takara, Kyoto, Japan). The mRNA expression of target genes was examined by a standard SYBR Green system on an ABI PRISM 7500 sequence detection system (Applied Biosystems, Foster City, CA, USA). The primer sequences are shown in Supplement Table 1. The qRT-PCR conditions were as follows: 95°C for 30 s, followed by 40 cycles of denaturation at 95°C for 15 s and annealing and extension at 60°C for 60 s. All results were normalized to the expression level of *β*-actin and quantified by the comparative (2^−ΔΔCt^) method.

### 2.12. Statistical Analysis

Statistical analyses and figure formatting were conducted using the SPSS 24.0 software (Vision 24.0, IBM, Armonk, NY, USA) and GraphPad Prism software (Vision 8.0, GraphPad Software, San Diego, CA, USA), respectively. Data were expressed as the mean ± standard deviation. Data were statistically evaluated via one-way ANOVA followed by the LSD *t* test. *P* < 0.05 was considered statistically significant.

## 3. Results

### 3.1. Isolation of RC

For the first time, we extracted, separated, and identified RC from Rn produced in China. After enrichment and elution by strongly basic styrene anion exchange resin (Amberlite TRA-67), the HPLC chromatogram of the eluent was generated (Supplement Figure 1A). The corresponding components were collected, and the orange-red oily residue (25 g) was recovered under reduced pressure. RC (Anna Sendl et al., 1996), C_25_H_30_O_5_, m/z 411.2080[M+H]^+^, collection (Supplement Figure 1B) was confirmed by NMR, and the NMR chromatogram of the eluent was generated (Supplement Figure 1C). The results showed that the Rn in China examined in this study and the Rn reported in Southeast Asia [21, 22] have similar chemical components, both of which contain a high content of RC.

### 3.2. Molecular Docking

**Figure 1:**
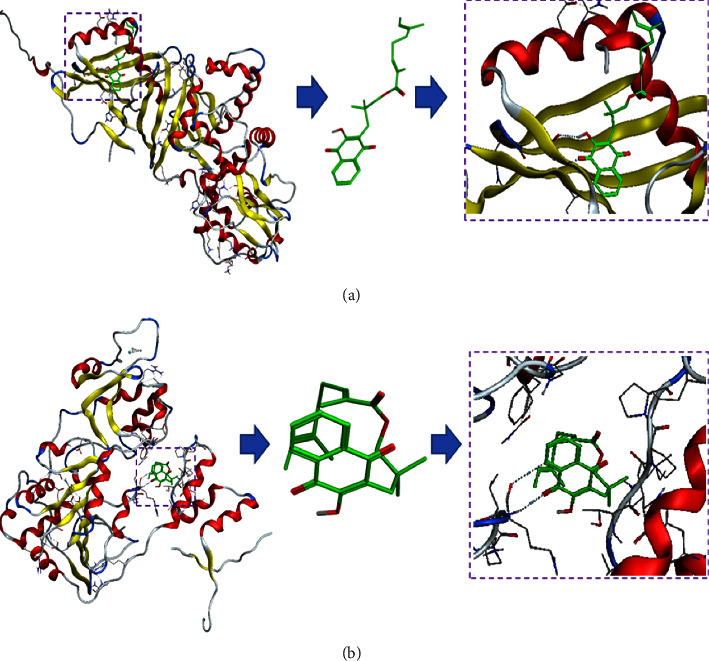
Molecular docking results of RC with AMPK*α* and SIRT1. (a) 3D binding model of RC with the molecular surface of AMPK*α* coloured in grey (2ya3). (b) 3D binding model of RC with the molecular surface of SIRT1 coloured in grey (5btr).

To reveal the binding mode and affinity of RC with AMPK*α* and SIRT1, molecular docking was performed. Docking scores showed similar binding affinity of RC with both protein kinases related to the liver fat metabolism (–5.63 and –3.93 kj/mol). Figures 1(a) and 1(b) both show that RC bound to the surface cavity of AMPK*α* and SIRT1, while the moiety of RC outside of the protein binding pocket was different. The 2D binding mode with AMPK*α* showed that the N-H-*π* interaction between His397 and the benzene ring and the interaction between the carbonyl on RC and the hydroxyl on the naphthalene ring involve hydrogen bonds with Arg401, and both of these interactions played major roles in the binding affinity of RC for AMPK*α*. In addition, the benzene ring is in a hydrophobic hole formed by Lle543, Val549, Thr548, and so on, indicating that hydrophobic interactions may also be an important factor in the binding affinity of RC for AMPK*α*. The 2D binding mode with SIRT1 shows that the carbonyl on RC has a hydrogen bond interaction with Phe414, which plays a major role in the binding affinity. However, due to the internal folding of olefin's long chain, its hydrophobicity reduces the role of hydrogen bonds, and the hydroxyl is wrapped inside the molecule, which prevents the hydroxyl from forming a hydrogen bond. In addition, the naphthalene ring is in a hydrophobic hole formed by Arg446, Val445, Pro212, Gly415, and so on, indicating that hydrophobic interactions may also be an important factor in the binding affinity of RC for SIRT1.

### 3.3. RC Prevents HFD-Induced Obesity in Mice

**Figure 2:**
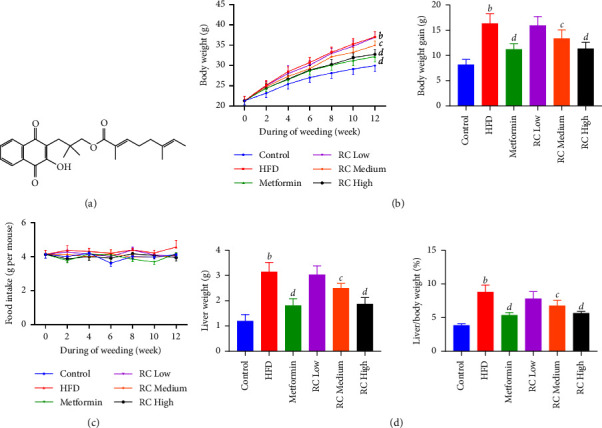
RC prevents HFD-induced obesity in mice. (a) Chemical structure of RC. (b) Body weight and body weight gain of mice in each group were monitored for 12 weeks. (c) The food intake of mice in each group was monitored for 12 weeks. (d) Liver weight and liver weight gain of mice in each group were monitored for 12 weeks. Values (mean ± SEM, *n* = 12). ^*b*^*P* < 0.01 vs. control group, ^*c*^*P* < 0.05, and ^*d*^*P* < 0.01 vs. HFD group.

The chemical structure of RC is shown in Figure 2(a). Over 12 weeks, isolated RC was supplied to HFD-fed mice according to the dose used in a previous study [23]. To evaluate the effect of RC on obesity, body weight was monitored every 2 weeks, and the body weight gain, liver weight, and liver/body weight ratio were calculated at the end of the feeding period. The results showed that HFD feeding significantly increased the body weight and liver weight of mice (*P* < 0.01). Interestingly, food intake did not significantly differ between the feeding groups (*P* > 0.05). Medium- and high-dose RC and metformin significantly inhibited body weight, liver weight, and the liver/body weight ratio of mice when compared with the HFD group (*P* < 0.05 and *P* < 0.01), which indicated that the trend for attenuation in HFD-induced obesity in the RC group was not due to a reduction in food intake (Figures 2(b)–2(d)).

### 3.4. RC Prevents HFD-Induced Hepatic Steatosis and Lipid Accumulation

**Figure 3:**
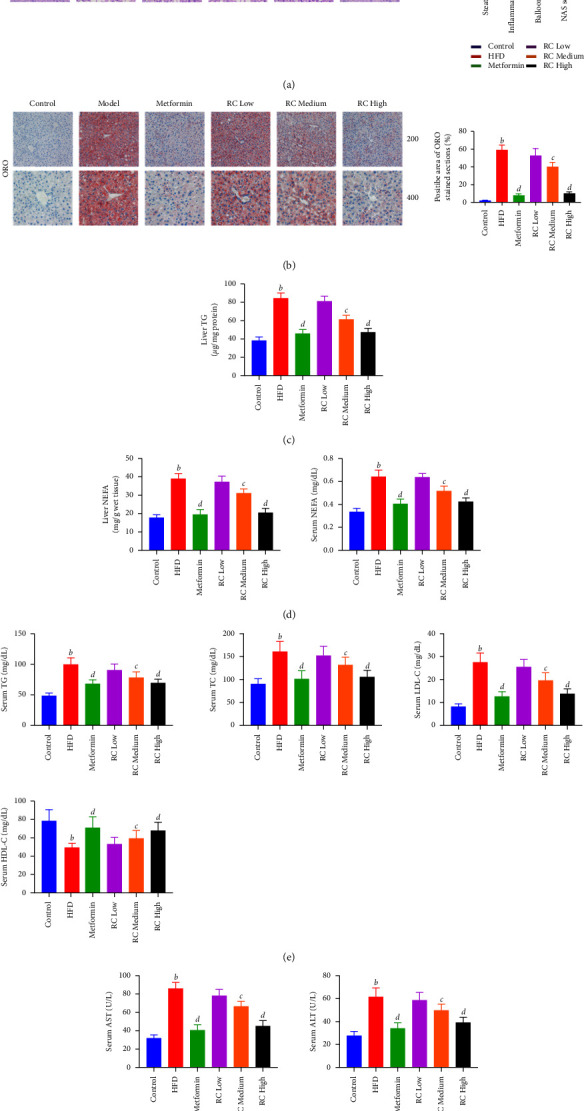
Inhibitory effects of RC prevent HFD-induced liver function, hepatic steatosis, and lipid accumulation in mice. The pathological features of liver tissue in each group were evaluated by H&E (a) and Oil Red O staining (b) (image magnification ×200&400). (c) Liver TG content was detected. (d) Liver NEFA and serum NEFA were analysed. (e) Serum TG, TC, LDL-C, and HDL-C contents were detected. (f) Serum AST and ALT levels were detected. The supernatant of tissue samples was analysed using the respective kits. Values (mean ± SEM, *n* = 6). ^*b*^*P*  <  0.01 vs. control group, ^*c*^*P* < 0.05, and ^*d*^*P* <  0.01 vs. HFD group.

Histopathological examination showed that HFD-induced steatosis, inflammation and ballooning, and NAS score [24] were significantly increased compared with those of the control group (*P* < 0.01). Compared with the HFD group, metformin and medium- and high-dose RC significantly attenuated these liver changes and reduced NAS scores (*P* < 0.01) (Figure 3(a)). Consistent with the histopathological analysis, HFD feeding resulted in an increase in serum biochemical levels of AST and ALT (*P* < 0.01). RC treatment inhibited the increase in AST and ALT levels induced by HFD in a dose-dependent manner (*P* < 0.05 and *P* < 0.01) (Figure 3(f)).

Lipid accumulation in hepatocytes was observed by ORO staining. A large amount of bright orange lipid deposits was observed after 12 weeks of feeding, and the positive staining area was significantly increased compared with that of the control group (*P* < 0.01) (Figure 3(b)). Levels of TG and NEFA in the liver tissue and NEFA, TG, TC, and LDL-C in the serum were also increased (*P* < 0.01), and the level of HDL-C in serum was decreased in the HFD group (*P* < 0.01). After metformin treatment, the lipid accumulation in hepatocytes was significantly lower than that in the HFD group, the levels of T and NEFA in the liver tissue and NEFA, TG, TC, and LDL-C in serum were decreased (*P* < 0.01), and the level of HDL-C in serum was increased (*P* < 0.01). RC treatment showed the same trend as metformin and inhibited lipid deposition in hepatocytes in a dose-dependent manner (*P* < 0.05 and *P* < 0.01) (Figures 3(b)–3(e)). Collectively, these data suggest that RC may reduce the liver injury by reducing liver lipid accumulation in mice.

### 3.5. RC Improves HFD-Induced Insulin Resistance in Mice

**Figure 4:**
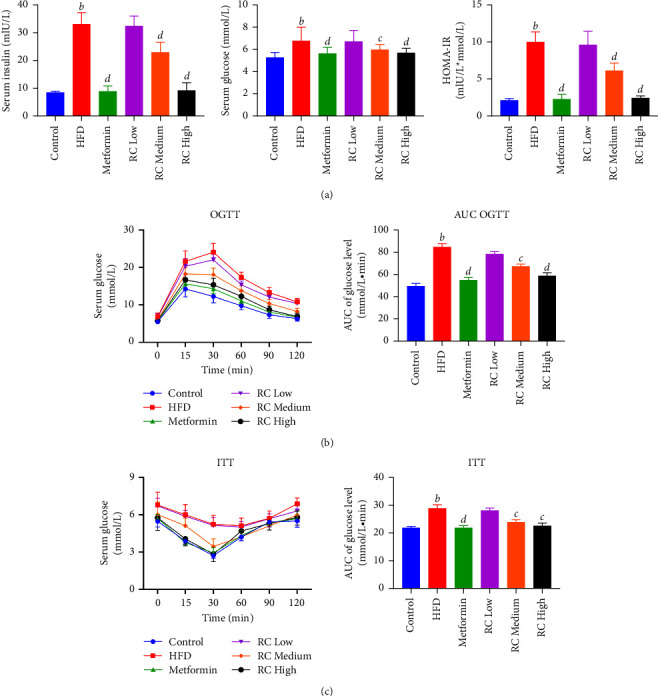
RC decreased HFD-induced insulin resistance in mice. After RC treatment, the tissue samples were analysed using the respective kits. (a) Serum insulin, serum glucose, and HOMA-IR content were detected. Increases in the OGTT (b) and ITT values (c) were observed. Values (mean ± SEM, *n* = 6). ^*b*^*P*  <  0.01 vs. control group, ^*c*^*P*  <  0.05, and ^*d*^*P*  <  0.01 vs. HFD group.

**Figure 5:**
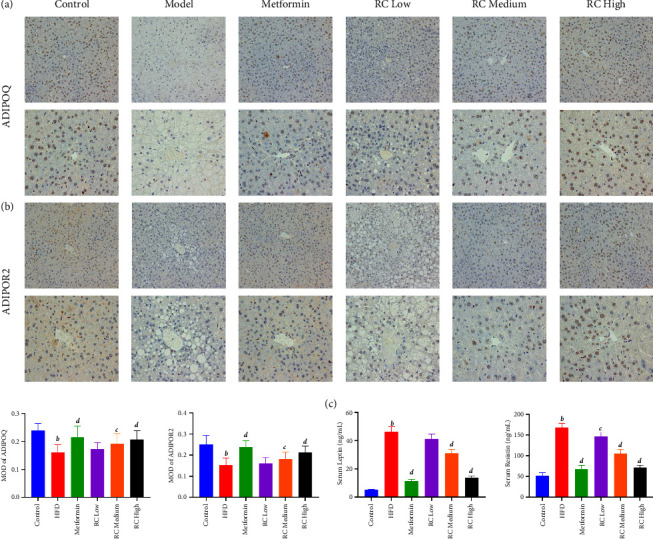
RC decreased HFD-induced lipid accumulation by IMC. The pathological features of liver tissue in each group were evaluated using ADIPOQ (a) and ADIPOR2 (b) by IMC (image magnification ×200 & 400), (c) MOD of ADIPOQ and ADIPOR were analysed, and serum leptin and serum resistin were detected. Values (mean ± SEM, *n* = 6). ^*b*^*P*  <  0.01 vs. control group, ^*c*^*P*  <  0.05, and ^*d*^*P*  <  0.01 vs. HFD group.

Compared with the control group, HFD feeding significantly increased the serum levels of insulin, glucose, leptin, and resistin (*P* < 0.01) and significantly decreased the levels of ADIPOQ and ADIPOR2 in the liver tissue (*P* < 0.01). We also observed elevations in HOMA-IR, OGTT, and ITT values in the HFD group. When compared with the HFD group, ADIPOQ and ADIPOR2 levels in the liver tissue were increased and serum levels of insulin, glucose, leptin, and resistin and HOMA-IR, OGTT, and ITT values were decreased in the medium- (10 mg/kg) and high-dose (20 mg/kg) RC groups, with the greatest difference seen in the high-dose RC group (Figures 4 and 5).

### 3.6. RC Improves HFD-Induced Inflammation in Mice

**Figure 6:**
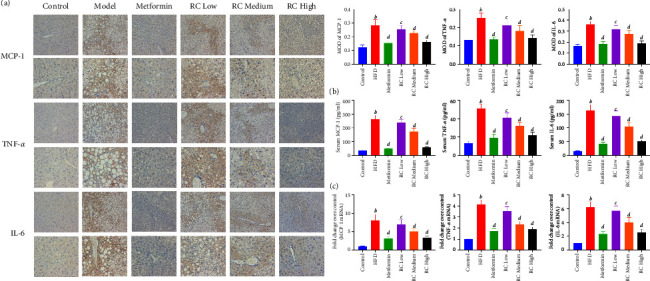
RC decreased HFD-induced inflammation by IMC. The mRNA level of ANGPTL8 was measured by RT‒qPCR. (a) The pathological features of liver tissue in each group were evaluated for MCP-1, TNF-*α*, and IL-6 by IMC (image magnification ×200 & 400). (b) MODs of MCP-1, TNF-*α*, and IL-6 were analysed, and serum MCP-1, TNF-*α*, and IL-6 were detected. (c) The fold changes over the control of MCP-1, TNF-*α*, and IL-6 were detected. Values (mean ± SEM, *n* = 4). ^*b*^*P*  <  0.01 vs. control group, ^*c*^*P*  <  0.05, and ^*d*^*P*  <  0.01 vs. HFD group.

Next, we evaluated the effect of RC on the HFD-induced inflammatory response.  Figure 6 shows that HFD feeding significantly increased the levels of MCP-1, TNF-*α,* and IL-6 in the liver and serum when compared with the control group (*P* < 0.01). However, RC administration attenuated these increases in a dose-dependent manner (medium-dose RC group, *P* < 0.05; high-dose RC group, *P* < 0.01). These data demonstrate that RC reduced the HFD-induced inflammatory response.

### 3.7. RC Activates AMPK/SIRT1/NF-Κb Pathway

**Figure 7:**
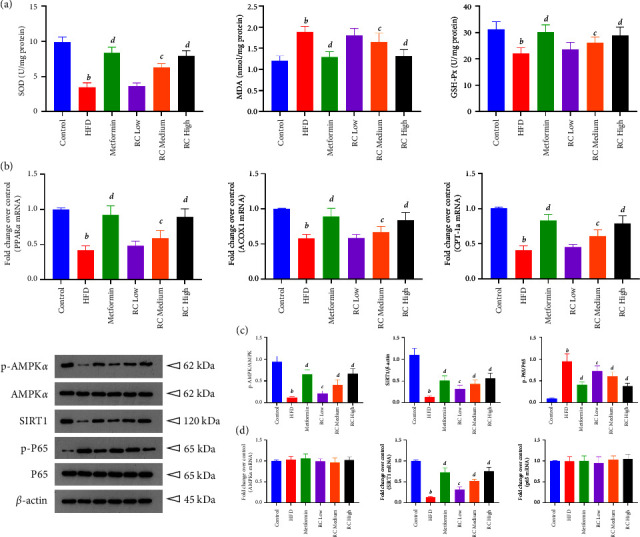
RC activates the AMPK/SIRT1/NF-*κ*B pathway. After RC treatment, the tissue samples were analysed by ELISA and RT‒qPCR, and protein expression was subjected to Western blot analysis. (a) SOD, MDA, and GSH-Px were detected by ELISA. (b) Effect of RC on PPAR*α*, ACOX1, and CPT-1A protein expression. (c) Representative Western blot results of p-AMPK*α*, AMPK*α*, SIRT1, p-P65, and P65 in mouse liver tissues and densitometric analysis of p-AMPK*α*, AMPK*α*, SIRT1, p-P65, and P65. Values were normalized to that of *β*-actin. (d) Fold changes over control of AMPK*α*, SIRT1, and P65 were analysed. Values (mean ± SEM, *n* = 4). ^*b*^*P*  <  0.01 vs. control group, ^*c*^*P*  <  0.05, and ^*d*^*P*  <  0.01 vs. HFD group.

HFD induced an increase in SOD and GSH-Px activities and a decrease in MDA concentration (*P* < 0.01). Furthermore, HFD feeding significantly downregulated the expression of fatty acid oxidation markers, including PPAR*α*, ACOX1, and CPT-1*α*, when compared with the control group (*P* < 0.01). RC administration dose-dependently decreased the activities of SOD and GSH-Px, increased the concentration of MDA, and upregulated the mRNA expression levels of PPAR*α*, ACOX1, and CPT-1*α* in the liver tissue when compared with the HFD group (*P* < 0.01) (Figures 7(a) and 7(b)).

To further determine whether the AMPK/SIRT1/NF-*κ*B pathway was involved in the RC-induced antioxidative effect, we examined the phosphorylation of related proteins and the expression levels of the associated mRNAs in liver tissue.  Figure 7(c) shows that the levels of p-AMPK*α* and Sirt1 were significantly reduced, and the level of p-NF-кB p65 was significantly increased (*P* < 0.01). Compared with the HFD group, the metformin group and the low-, medium- and high-dose RC administration groups showed dose-dependent upregulation of p-AMPK*α* and Sirt1 and downregulation of p-NF-*κ*B p65 in the liver tissue (*P* < 0.05 and *P* < 0.01). The same trends were also observed for hepatic SIRT1 mRNA expression (*P* < 0.05 and *P* < 0.01), while no significant differences were found in the mRNA expression levels of AMPK*α* or NF-кB p65 (*P* > 0.05) (Figure 7(d)).

### 3.8. RC Inhibits SREBP-1c/FAS/ACC Pathway

**Figure 8:**
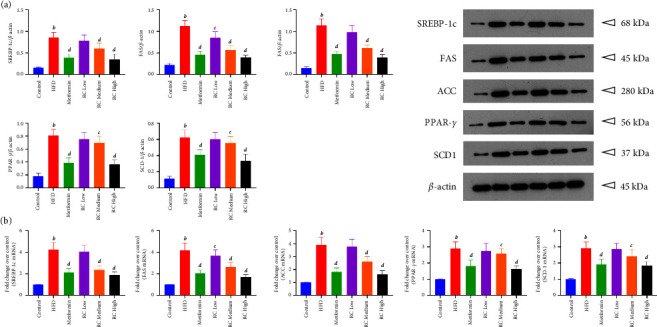
RC activates the SREBP-1c/FAS/ACC pathway. After RC treatment, the protein levels of SREBP-1c, FAS, ACC, PPAR-*γ*, and SCD1 were examined by Western blot analysis. (a) Representative Western blot results of SREBP-1c, FAS, ACC, PPAR-*γ*, and SCD1 in mouse liver tissues and densitometric analysis of SREBP-1c, FAS, ACC, PPAR-*γ*, and SCD1. Values were normalized to that of *β*-actin. (b) Fold changes over control of SREBP-1c, FAS, ACC, PPAR-*γ*, and SCD1 were analysed. Values (mean ± SEM, *n* = 4). ^*b*^*P*  <  0.01 vs. control group, ^*c*^*P*  <  0.05, and ^*d*^*P*  <  0.01 vs. HFD group.

Concurrently, we detected the expression levels of related proteins and mRNA in the SREBP-1c/Fas/ACC pathway in the liver tissue (Figure 8). HFD feeding significantly upregulated the expression levels of SREBP-1C, FAS, ACC, PPAR-*γ*, and SCD1 in the liver tissue (*P* < 0.01). After metformin treatment, the expression levels of SREBP-1C, FAS, ACC, PPAR-*γ*, and SCD1 were significantly downregulated when compared with the HFD group (*P* < 0.01), with RC administration showing the same trend in a dose-dependent manner (*P* < 0.05 and *P* < 0.01). These data suggest that RC inhibited the activation of the SREBP-1c/Fas/ACC signaling pathway.

## 4. Discussion

The rising prevalence and severity of NAFLD are closely associated with the obesity epidemic, which has been linked not only to simple hepatic steatosis but also to nonalcoholic steatohepatitis (NASH), NASH-related cirrhosis, and hepatocellular carcinoma (HCC) [25, 26]. A previous 12-year prospective Japanese study showed that BMI increase was associated with the onset of NAFLD, whereas BMI decrease was associated with NAFLD resolution, notably, in both obese and nonobese individuals [27]. In particular, hepatic de novo lipogenesis is the primary mechanism for developing and maintaining hepatic steatosis in individuals with obesity and NAFLD, and weight loss decreases intrahepatic triglyceride contents by decreasing hepatic lipogenesis, thereby rendering lipogenesis a potential therapeutic target for NAFLD. As a consequence, apart from increasing all-cause mortality, obesity seems to increase liver-specific mortality in NAFLD patients [28, 29]. Our results showed that HFD induced a significant increase in the weight of mice and RC significantly inhibited the weight gain and visceral obesity of NAFLD mice without affecting food intake and reduced the liver index (liver/body weight) in a dose-dependent manner.

NAFLD is also characterized by elevated levels of LDL-C, TG, ALT, AST, and NEFA and by decreased HDL-C concentrations [30]. In the present study, the hepatocytes were steatosis obviously in the model group, and RC reduced the degree of hepatocyte steatosis and the number of intercellular vacuoles as expected. The serum levels of LDL-C, TG, ALT, AST, and NEFA were significantly increased, while HDL-C concentrations significantly were decreased in the model group. The changes mentioned previously were significantly ameliorated by the RC treatment, suggesting that RC is conducive to improving the liver function level of NAFLD mice, thereby improving the hyperlipidemia and liver injury of NAFLD mice.

Insulin resistance refers to the decreased sensitivity of target organs to the action of insulin and excessive fatty acids in the circulation, which lead to simple hepatic steatosis, forming the first hit of NAFLD [31]. Both leptin and resistin are secreted proteins, and the levels of leptin and resistin increase when high-fat and high-sugar foods are ingested and in response to other factors, leading to insulin resistance. In this study, we demonstrated that RC significantly reduced insulin and glucose levels, thereby ameliorating HFD-induced insulin resistance. Meanwhile, the RC treatment also significantly reversed the increases in ADIPOQ, ADIPOR2, leptin, and resistin caused by HFD.

The second hit involves oxidative stress, lipid peroxidation, and mitochondrial dysfunction [5]. Hepatic steatosis, a hallmark feature of NAFLD, results from increased de novo lipogenesis, fat-derived fatty acid flux, and impaired triglyceride secretion. Briefly, ACC1 catalyses the conversion of acetyl-CoA to malonyl-CoA; this is the first rate-limiting step in the fatty acid synthesis. Malonyl-CoA is then catalysed by the main biosynthetic enzyme FAS to generate palmitate [32, 33]. De novo lipogenesis is a complex and highly regulated metabolic pathway wherein fatty acids are synthesized from carbohydrates or amino acids and then incorporated into triglycerides or other lipid molecules. FAS and ACC are important rate-limiting enzymes for fatty acid synthesis. Finally, palmitic acid formed under a series of enzymatic actions undergoes elongation and desaturation to generate complex fatty acids, including oleic and palmitoleic acids. The activation of SREBP-1C enhances fatty acid synthesis and accelerates TG accumulation, which plays an important role in NAFLD. In addition, PPAR-*γ* and SCD1 enzymes are the main substrates for the synthesis of triglycerides and other lipids. In NAFLD, PPAR-*γ* is upregulated in the liver tissue, and liver-specific PPAR-*γ* mice are protected from diet-induced steatosis [34]. In this study, our results showed that RC significantly downregulate the expression levels of SREBP-1C, FAS, ACC, PPAR-*γ*, and SCD1 in liver tissue, inhibit the hepatic de novo lipogenesis, and reduce fat derivation and triglyceride secretion, thereby inhibiting lipid accumulation.

Oxidative stress can cause inflammatory infiltration of cells, increase the secretion of protease, and produce a large number of oxidation intermediates. Lipid peroxidation is known as the second hit of NAFLD [35]. Oxidative stress and lipid peroxidation both involve a series of ROS-induced cytotoxic events in liver parenchyma cells. In the present study, our findings showed that RC significantly upregulated the expression levels of PPAR-*γ*, ACOX1, and CPT1*α* in liver tissue, thereby improving the fatty acid *β*-oxidation ability. Meanwhile, RC administration dose-dependently decreased the activities of SOD and GSH-Px, increased the concentration of MDA, and inhibited the oxidative stress level of NAFLD. An excessive inflammatory response is one of the typical pathological changes in NAFLD, and inflammatory factors, such as TNF-*α* and IL-6, are involved in the secondary liver injury of NAFLD and can even cause necrosis and fibrosis. MCP-1 is a specific chemokine that promotes the migration of monocytes from the peripheral circulation to the site of inflammation to aggregate and activate them and damage the body tissue through a variety of mechanisms. The present findings showed that RC significantly inhibited the levels of MCP-1, TNF-*α,* and IL-6, thereby decreasing the level of liver inflammation in NAFLD. Interestingly, Zhao et al. [12] found that RC can increase renal levels of glutathione, superoxide dismutase, and catalase and attenuate diabetic-induced renal damages, protective effect against DN through the inhibition of the inflammatory damage caused by oxidative stress.

AMPK, a vital sensor activated in response to stress in the regulation of cellular energy homeostasis, plays an essential role in the lipid, energy, and carbohydrate metabolism of hepatocytes and is also a promising key player in curing obesity and NAFLD [36]. AMPK can produce anti-inflammatory effects and inhibit oxidative stress through the activation of the SIRT1/NF-*κ*B pathway. SIRT1 plays a key role in the development of NAFLD by participating in the regulation of lipid and carbohydrate metabolism in liver tissue [32]. The PPAR-*γ* also has anti-inflammatory features as it represses NF-*κ*B signaling [37]. Adipose de novo synthesis is an important source of liver fat deposition. Several studies have shown that AMPK is a receptor regulating cell energy metabolism. The AMPK/SIRT1 pathway is involved in the synthesis, metabolism, and output of fatty acids and glucose in the body and is one of the key links in NAFLD. The activation of AMPK can increase catabolism, reduce the expression levels of ACC, FAS, SREBP, and other lipid synthesis-related factors, and reduce the rate of anabolism, regulating the synthesis, and utilization of lipids. SIRT1 further controls downstream lipid regulators by mediating the role of AMPK in an LKB1-dependent manner. The overexpression of SIRT1 can also enhance the phosphorylation of AMPK and ACC, further inhibiting the increase in FFA and the accumulation of blood lipids induced by high glucose [38]. The two-hit-hypothesis has been proposed to explain the pathogenesis of NASH [39]. Our results showed that the protein levels of phosphorylated AMPK*α* and SIRT1 were significantly inhibited in liver tissues of NAFLD mice, further activating phosphorylation of NF-*κ*B p65. RC intervention significantly reversed AMPK/SIRT1/NF-*κ*B activation, thereby decreasing NAFLD fatty acid *β* oxidation, oxidative stress, and inflammation. However, the mechanisms underlying the pathogenesis and progression of NAFLD are still incompletely understood and NAFLD was shown to develop through a multifactorial process including genetic determinants, nutrition and lifestyle, and changes in the intestinal microbiota [40].

## 5. Conclusions

**Figure 9:**
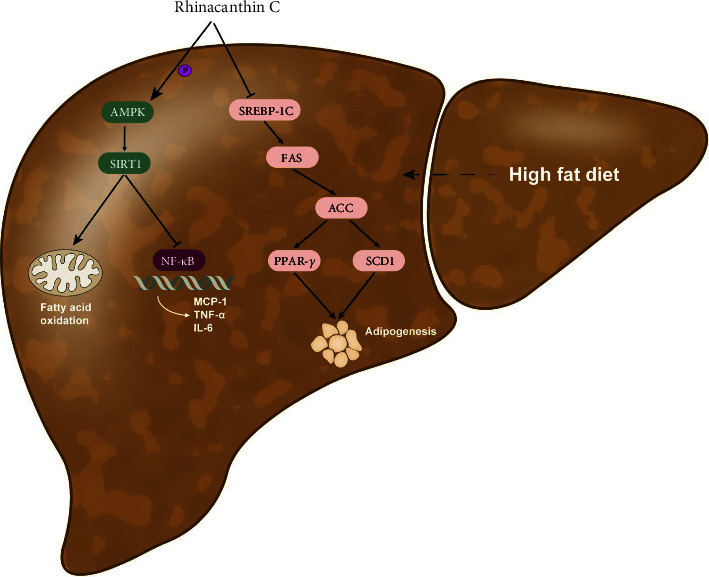
Summary of the study. *Rhinacanthin C* upregulated the levels of AMPK and SIRT1. High SIRT1 expression reduced MCP-1, TNF-*α*, and IL-6 activity, which might induce fatty acid oxidation and alleviate inflammatory injury. In addition, RC decreased nucleic SREBP-1c and ACC activity, which might suppress lipogenesis in the liver. Thus, RC ameliorates insulin resistance and lipid accumulation in NAFLD mice via the AMPK/SIRT1 and SREBP-1c/FAS/ACC signaling pathways.

The results of the present study revealed that long-term consumption of HFD will lead to continuous obesity and glucose tolerance, leading to histopathological abnormalities and lipid metabolism disorders of nonalcoholic fatty liver. Oral administration of RC could protect against NAFLD development in HFD-fed mice in a dose-related manner, as evidenced by reducing the weight and glucose level of HFD mice, improving liver physiological changes, and restoring metabolic markers of NAFLD.  Figure 9 shows that the compound may reverse AMPK/SIRT1/NF-*κ*B activation, thereby reversing the increases in ADIPOQ, ADIPOR2, leptin, insulin, glucose levels, and resistin caused by HFD and decreasing NAFLD fatty acid *β* oxidation, oxidative stress, and inflammation, which inhibit lipid accumulation and improve the level of liver inflammation in NAFLD. In the present study, RC could be used for preventing and managing of Western diet-related chronic diseases, including type 2 diabetes mellitus and NAFLD.

## Data Availability

The data used to support the findings of this study are available from the corresponding author upon request.

## Supplementary Materials

Supplementary Materials Supplement Table 1: primers used in quantitative real-time PCR reactions. Supplement Figure 1: extraction, isolation, and structural identification of RC. (A) HPLC chromatograms of the R. nasutus rhizome ethyl acetate extract; the mobile phase was MeOH/0.2% TFA/H2O (82 : 18, v/v); and the quantification wavelength was set at 250 nm. (B) Experimental IDA TOF MS of RC (M + H)^+^. (C) ^1^H NMR and ^13^C NMR spectra of RC.
