# Supplementary material for: Rhinacanthin C Ameliorates Insulin Resistance and Lipid Accumulation in NAFLD Mice via the AMPK/SIRT1 and SREBP-1c/FAS/ACC Signaling Pathways
Source: Evid Based Complement Alternat Med. 2023 Jan 10;2023:6603522. doi: 10.1155/2023/6603522 (PMC9845057; doi:10.1155/2023/6603522)

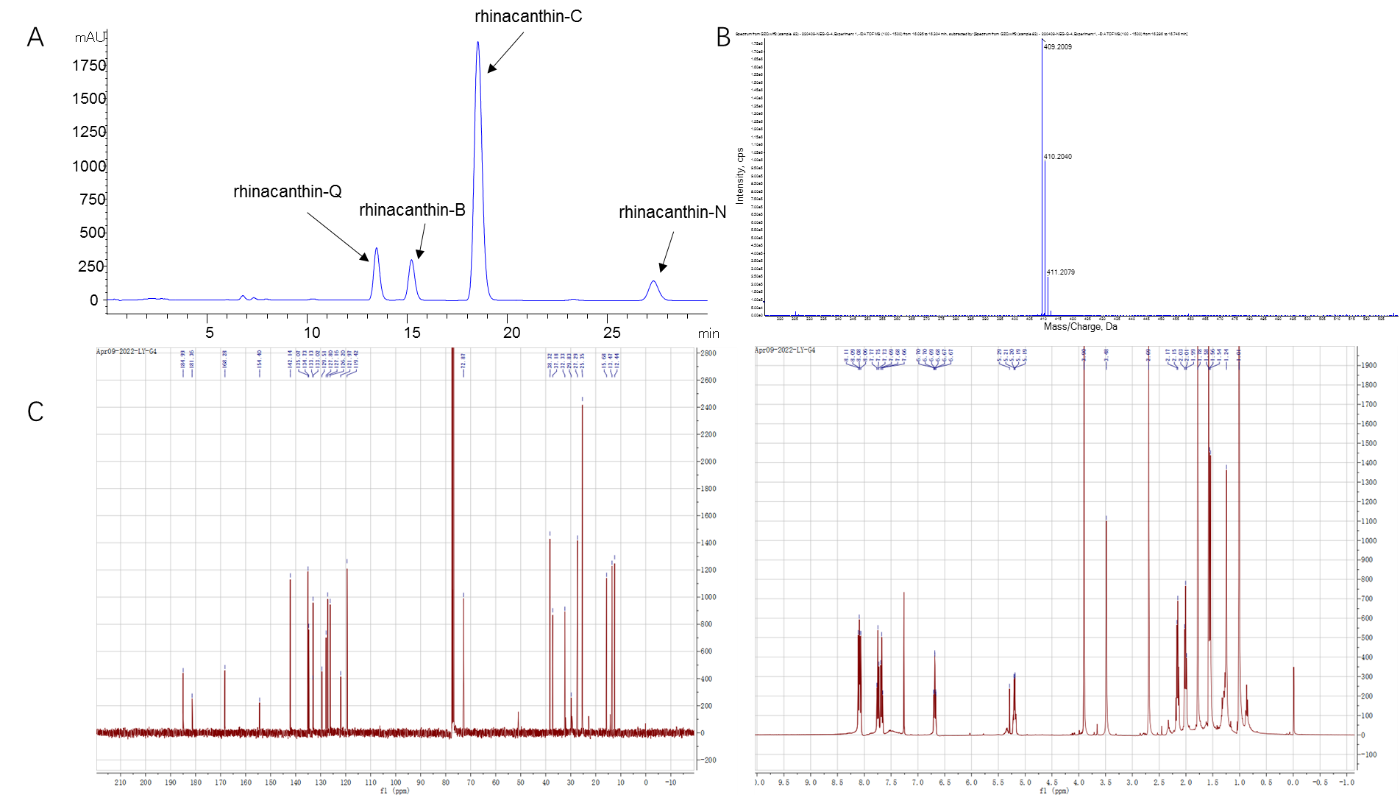


Supplement Figure 1: Extraction, isolation and structural identification of RC. (A) HPLC chromatograms of the R. nasutus rhizome ethyl acetate extract; the mobile phase was MeOH/0.2% TFA/H2O (82:18, v/v) and the quantification wavelength was set at 250 nm. (B) Experimental IDA TOF MS of RC (M+H)^+^, (C) ^1^H NMR and ^13^C NMR spectra of RC.


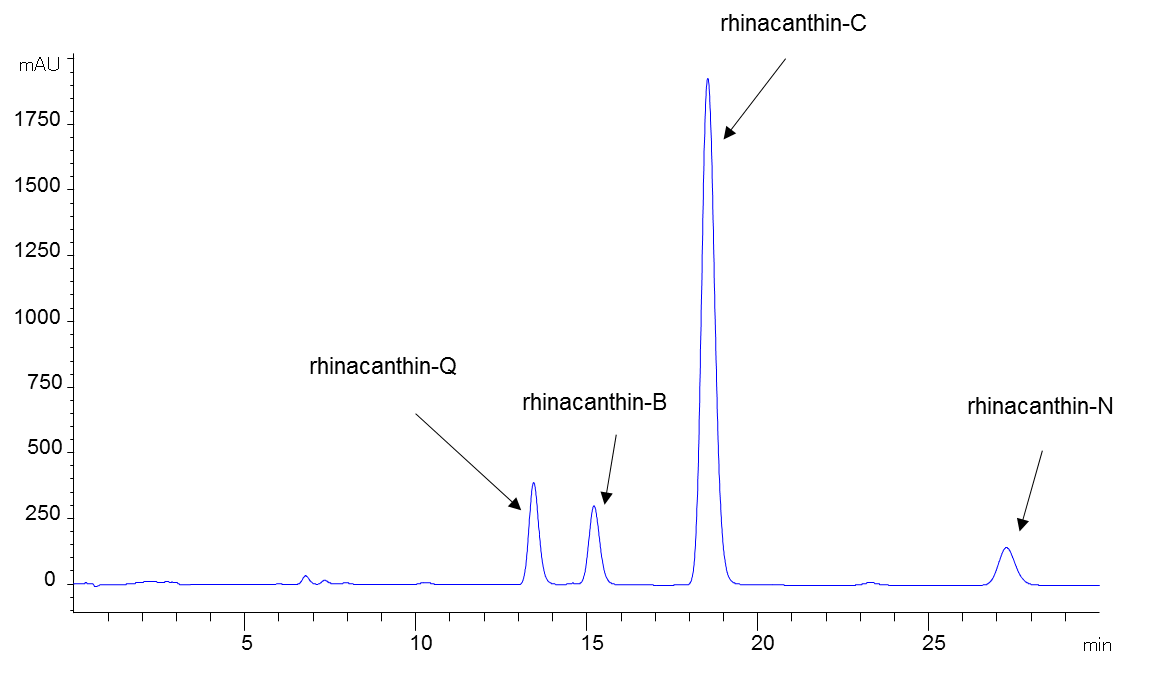


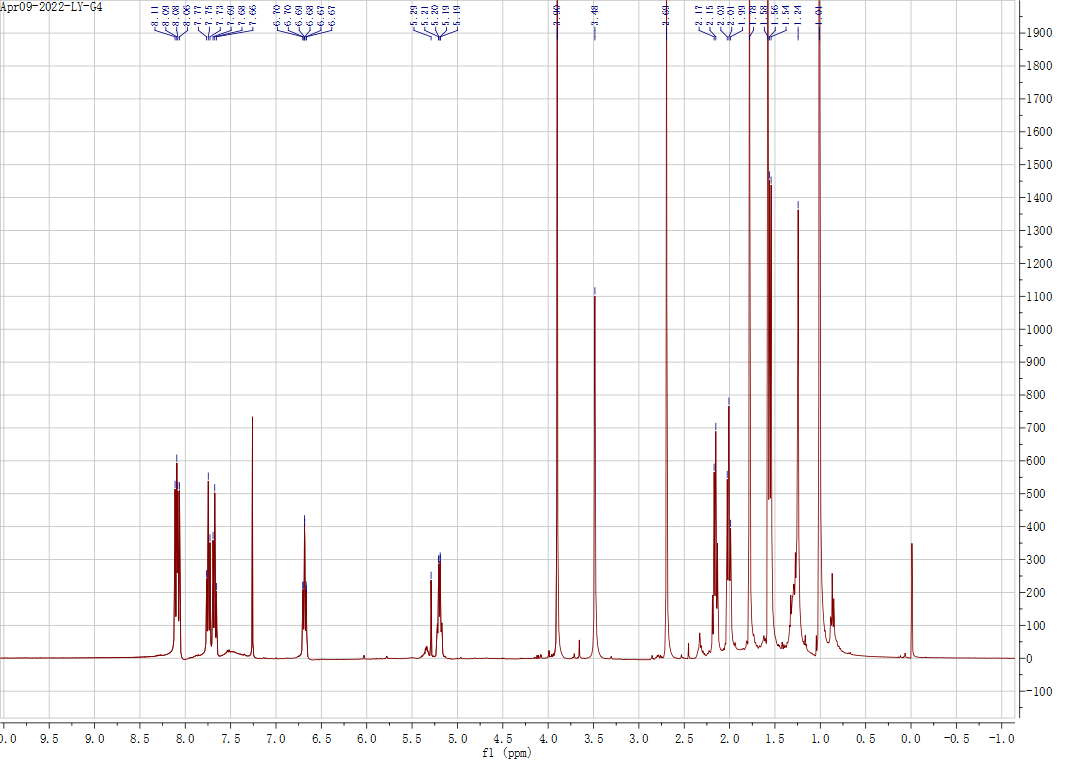


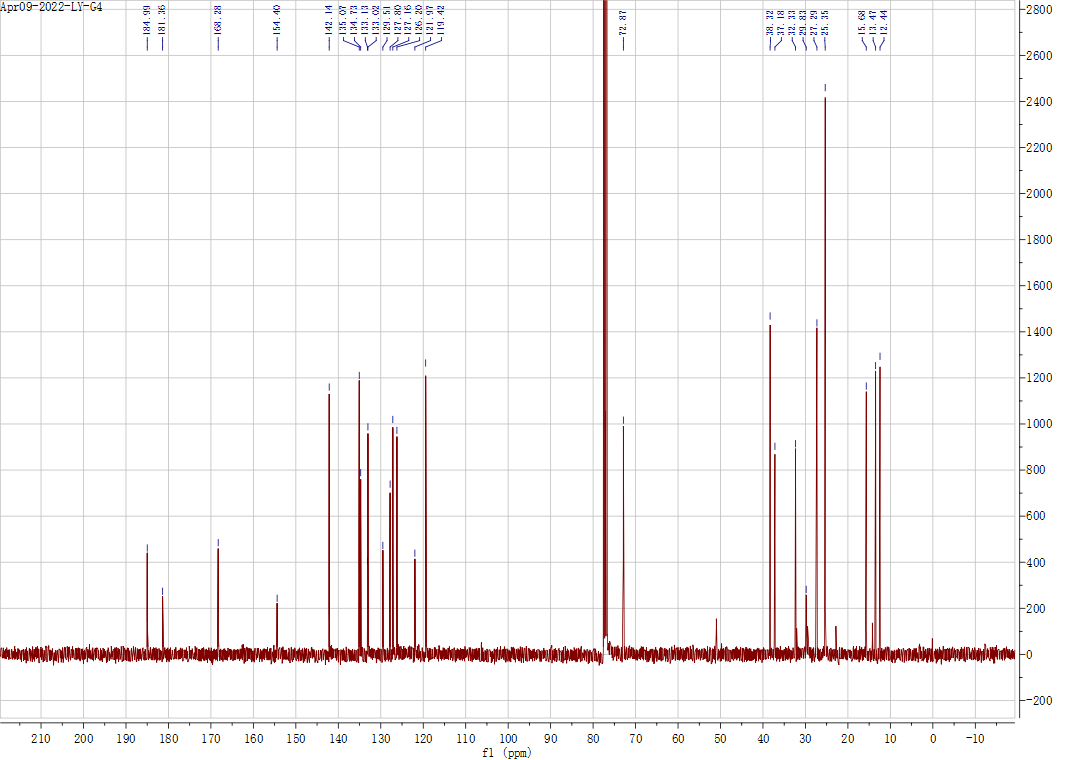

Supplement: Supplementary Materials — Supplement Table 1: primers used in quantitative real-time PCR reactions. Supplement Figure 1: extraction, isolation, and structural identification of RC. (A) HPLC chromatograms of the R. nasutus rhizome ethyl acetate extract; the mobile phase was MeOH/0.2% TFA/H2O (82 : 18, v/v); and the quantification wavelength was set at 250 nm. (B) Experimental IDA TOF MS of RC (M + H)+. (C) 1H NMR and 13C NMR spectra of RC. [file 6603522.f1.zip › Supplementary Fig.1.docx]
