# Supplementary material for: Rhinacanthin C Ameliorates Insulin Resistance and Lipid Accumulation in NAFLD Mice via the AMPK/SIRT1 and SREBP-1c/FAS/ACC Signaling Pathways
Source: Evid Based Complement Alternat Med. 2023 Jan 10;2023:6603522. doi: 10.1155/2023/6603522 (PMC9845057; doi:10.1155/2023/6603522)
Supplement: Supplementary Materials — Supplement Table 1: primers used in quantitative real-time PCR reactions. Supplement Figure 1: extraction, isolation, and structural identification of RC. (A) HPLC chromatograms of the R. nasutus rhizome ethyl acetate extract; the mobile phase was MeOH/0.2% TFA/H2O (82 : 18, v/v); and the quantification wavelength was set at 250 nm. (B) Experimental IDA TOF MS of RC (M + H)+. (C) 1H NMR and 13C NMR spectra of RC. [file 6603522.f1.zip › Supplementary Table.1.docx]

Supplementary Table 1 Primers used in quantitative real-time PCR reactions.

**Table1. Primer sequences.**

| **Gene** | **Primers** | **Sequence (5′ to 3′)** |
| --- | --- | --- |
| MCP-1 | Forward | 5′-GCATCCACGTGTTGGCTCA-3′ |
|  | Reverse | 5′-CTCCAGCCTACTCATTGGGATCA-3′ |
| TNF-α | Forward | 5′- CTGAACTTCGGGGTGATCGG-3′ |
|  | Reverse | 5′- CTGAACTTCGGGGTGATCGG-3′ |
| IL-6 | Forward | 5′-CTGCAAGAGACTTCCATCCAG-3′ |
|  | Reverse | 5′-AGTGGTATAGACAGGTCTGTTGG-3′ |
| PPARα | Forward | 5′- TACTGCCGTTTTCACAAGTGC -3′ |
|  | Reverse | 5′- AGGTCGTGTTCACAGGTAAGA -3′ |
| ACOX1 | Forward | 5′- TAACTTCCTCACTCGAAGCCA-3′ |
|  | Reverse | 5′- AGTTCCATGACCCATCTCTGTC-3′ |
| CPT-1a | Forward | 5′- TGGCATCATCACTGGTGTGTT-3′ |
|  | Reverse | 5′- GTCTAGGGTCCGATTGATCTTTG-3′ |
| AMPKα | Forward | 5′- TACTCAACCGGCAGAAGATTCG-3′ |
|  | Reverse | 5′- AGACGGCGGCTTTCCTTTT-3′ |
| SIRT1 | Forward | 5′- ATGACGCTGTGGCAGATTGTT-3′ |
|  | Reverse | 5′- CCGCAAGGCGAGCATAGAT-3′ |
| P65 | Forward | 5′- TGCGATTCCGCTATAAATGCG-3′ |
|  | Reverse | 5′- TGCGATTCCGCTATAAATGCG-3′ |
| SREBP-1C | Forward | 5′- GCAGCCACCATCTAGCCTG -3′ |
|  | Reverse | 5′- CAGCAGTGAGTCTGCCTTGAT -3′ |
| FAS | Forward | 5′- GCGGGTTCGTGAAACTGATAA-3′ |
|  | Reverse | 5′- GCGGGTTCGTGAAACTGATAA-3′ |
| ACC | Forward | 5′- TGCAGATCTTAGCGGACCAA-3′ |
|  | Reverse | 5′- GCCTGCGTTGTACAGAGCAA-3′; |
| PPARγ | Forward | 5′- GGAAGACCACTCGCATTCCTT-3′ |
|  | Reverse | 5′- GTAATCAGCAACCATTGGGTCA-3′ |
| SCD1 | Forward | 5′- TTCTTGCGATACACTCTGGTGC-3′ |
|  | Reverse | 5′- CGGGATTGAATGTTCTTGTCGT-3′ |
| β-actin | Forward | 5′-GGAGATTACTGCCCTGGCTCCTAGC-3′ |
|  | Reverse | 5′-GGCCGGACTCATCGTACTCCTGCTT-3′ |
